# Training modality-specific differences in body composition, resting metabolic rate, diet, and gut microbial signatures in elite endurance and strength athletes

**DOI:** 10.3389/fnut.2026.1844692

**Published:** 2026-07-13

**Authors:** Gande Vennela, Vivekanand Upadhyay, Ravindranadh Palika, Karthikeyan Ramanujam, Mekam Maheshwar, Challa Suresh, Venkatesh Kaliaperumal

**Affiliations:** 1 ICMR-National Institute of Nutrition, Hyderabad, India; 2 Academy of Scientific and Innovative Research (AcSIR), Ghaziabad, India; 3 Akshat Seva Sadan, Patna, Bihar, India

**Keywords:** body composition, bone mineral density, endurance training, gut microbiota, nutrition, respiratory quotient, resting metabolic rate, strength training

## Abstract

**Background/objectives:**

The mode of athletic training exerts a profound influence on both physiological parameters and gut microbiota composition; yet, comprehensive integrative analyses in elite athletes are scarce. This study systematically profiled and contrasted body composition, bone health, resting metabolic rate (RMR), fasting respiratory quotient (RQ), hematological indices, dietary intake, and key gut bacterial taxa among elite Indian endurance athletes, strength athletes, and sedentary controls.

**Methods:**

Male participants were classified as endurance athletes (*n* = 27), strength athletes (*n* = 29), or sedentary controls (*n* = 24). Body composition and bone mineral density were measured by dual-energy X-ray absorptiometry (DEXA); RMR and RQ were measured *via* indirect calorimetry; hematological parameters and inflammatory markers were measured from fasting blood samples; dietary intake was assessed *via* 24-h recalls; and gut microbiota abundance was assessed using quantitative real-time polymerase chain reaction (qPCR).

**Results:**

Strength athletes had significantly greater body weight, lean mass, bone mineral density, and absolute RMR than both endurance athletes and controls. Endurance athletes exhibited lower body fat, elevated fasting fat oxidation, and hematological profiles indicative of enhanced oxygen transport. Athletes showed higher relative abundances of bile-tolerant and proteolytic taxa, including *Bacteroides* spp., *Prevotella*, and *Streptococcus* spp., compared with controls. Endurance athletes had higher relative abundance of *Methanobrevibacter*, whereas strength athletes exhibited higher abundance of *Desulphovibrio* spp. The relative abundance of *Lactobacillus* spp. was highest in controls, while *Fecalibacterium, Enterobacteriaceae, Bifidobacterium* spp., and *Akkermansia* did not differ between groups.

**Conclusions:**

Elite Indian endurance and strength athletes present distinctive, sport-specific physiological and gut microbial signatures. This integrative approach uncovers novel associations and underscores the complex interplay between training modality, nutrition, and the host–microbiome axis in elite athletic populations.

## 1 Introduction

Endurance and strength training elicit unique physiological adaptations tailored to the demands of their respective disciplines. Endurance training enhances aerobic capacity by improving oxygen delivery and utilization through increased mitochondrial density, skeletal muscle capillarization, and cardiovascular efficiency (1–3). In contrast, strength training primarily drives skeletal muscle hypertrophy, neuromuscular adaptation, and increased mechanical loading, thereby augmenting musculoskeletal development and bone health (4).

Beyond training modality, physiological characteristics are shaped by ethnicity, habitual diet, and environmental exposures. South Asian populations, particularly Indians, have lower lean body mass, reduced bone mineral density, and greater adiposity than Western cohorts at comparable BMI (5, 6). These population-specific traits may influence physiological adaptations and athletic performance, underscoring the need for context-specific evaluation.

Emerging evidence also implicates the gut microbiota in exercise metabolism, immune modulation, recovery, and overall performance (7). Exercise training has been linked to shifts in microbial diversity and the abundance of bacterial taxa relevant to energy metabolism and host regulation (8). However, gut microbial composition is profoundly shaped by ethnicity, diet, and host genetics, resulting in distinctive, population-specific microbiome profiles. Consequently, insights derived from Western athlete cohorts may not be directly transferable to Indian athletes.

Integrating physiological profiling with gut microbiota assessment offers a holistic lens for understanding sport-specific adaptations and host–microbiome interactions across training modalities. Yet, data on the interplay between physiological factors and gut microbial profiles in elite Indian athletes remain limited. Accordingly, this study combined comprehensive physiological characterization with targeted gut microbiota analysis in elite Indian endurance and strength athletes, benchmarking against sedentary controls. We hypothesized that endurance and strength athletes would exhibit distinct physiological and gut microbial signatures that reflect the metabolic and functional demands of their training regimens.

## 2 Materials and methods

### 2.1 Participants and study design

This cross-sectional study was conducted from October 2024 to September 2025 and included healthy adult male participants. As a cross-sectional study, the present investigation was designed to characterize modality-associated differences in physiological, dietary, and gut microbial profiles among participants; therefore, causal relationships cannot be established.

Ethical approval was granted by the Institutional Ethics Committee of ICMR–National Institute of Nutrition (ICMR-NIN), Hyderabad (IEC No. 23/08/2023). Written informed consent was obtained from all participants. Elite athletes were recruited primarily from the Elite Indian Centers, with supplementary recruitment from private academies. Sedentary controls were selected from ICMR-NIN PhD scholars and staff. Athlete classification followed the Mitchell system (9): endurance (high-dynamic) and strength (high-static). Endurance athletes participated in long-distance running, race walking, and badminton; badminton was included in this group because it is classified as a high-dynamic sport and requires sustained aerobic capacity, despite its intermittent nature (9, 10). Strength athletes were from weightlifting, shot put, discus throw, javelin throw, and hammer throw.

### 2.2 Inclusion and exclusion criteria

Male athletes aged 18–35 years were eligible for inclusion if they had completed a minimum of five consecutive years of structured, sport-specific training, participated in at least five training sessions per week (≥8 h per week), and had competed at the state or national level within the preceding year. Athletes who had participated in mixed-modality or team sports were excluded from the study. Sedentary controls were eligible if they were male, aged 18–35 years, engaged solely in activities of daily living, had not undertaken any structured exercise or organized sport in the previous year, and had a body mass index (BMI) within the range of 18.5–24.9 kg/m^2^. Subjects reporting acute or chronic illness, injury, and antibiotic use within the preceding 3 months were excluded from the study.

### 2.3 Data collection

**Figure 1:**
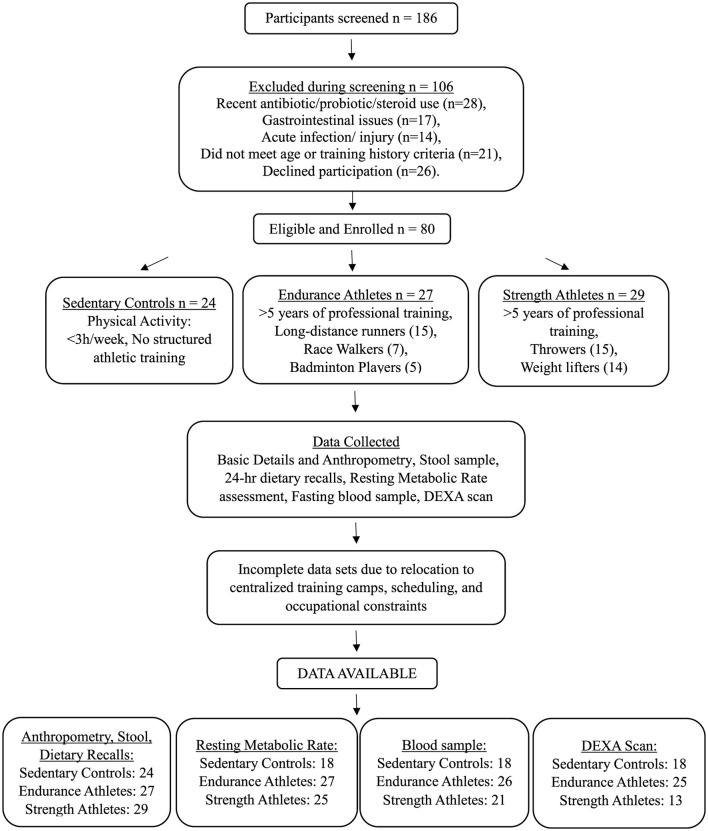
Flow diagram of participant screening, group allocation, and data availability, with exclusions, and incomplete datasets noted.

A total of 186 individuals were screened for eligibility; 106 were excluded due to ineligibility or refusal to participate (Figure 1). The final sample comprised 80 participants: 24 sedentary controls, 27 endurance athletes, and 29 strength athletes. All assessments were conducted at the ICMR–National Institute of Nutrition over five separate visits. At the initial visit, demographic information and training history were obtained, and eligibility was verified. The second visit, conducted 3–4 days later, included anthropometric measurements and dietary assessments. Participants received stool collection kits with instructions for sample collection and transportation. Fecal samples were subsequently returned to the institute for analysis. The third visit involved measurement of resting metabolic rate (RMR) and respiratory quotient (RQ) using indirect calorimetry. Fasting venous blood samples were collected during the fourth visit for hematological analysis. At the fifth visit, whole-body composition and bone mineral density were assessed using dual-energy X-ray absorptiometry (DEXA).

### 2.4 Anthropometry, body composition, and bone mineral density

Body mass and height were measured to the nearest 0.1 kg and 0.1 cm, respectively, by an ISAK-certified anthropometrist using calibrated Seca equipment. Whole-body composition and bone mineral density (BMD) were assessed by dual-energy X-ray absorptiometry (DEXA; Discovery A, Hologic Horizon, v13.3.0.1, USA). Participants attended the assessment after an overnight fast (8–12 h), maintained adequate hydration, and wore light clothing. Scans were conducted in the supine position with standardized limb placement, and site-specific BMD was measured at the lumbar spine (L1–L4), proximal femur, and forearm.

### 2.5 Resting metabolic rate and respiratory quotient

Participants reported to the Human Performance Laboratory at our institute after an overnight fast of at least 8 h, with abstention from caffeine or nicotine for at least 4 h and from structured physical activity for at least 12 h. RMR and RQ were assessed using a calibrated COSMED K5 metabolic system (11). Measurements were performed over 20 min, comprising a 5-min acclimation period followed by a 15-min steady-state phase, with participants resting supine in a quiet, temperature-controlled environment (22–25 C). RMR was calculated from oxygen consumption (VO_2_) and carbon dioxide production (VCO_2_) using Weir's equation, and RQ was calculated as VCO_2_/VO_2_. The COSMED K5 system was calibrated daily according to the manufacturer's guidelines, including flow calibration with a 3-L syringe and gas calibration with certified reference gases.

### 2.6 Hematological and biochemical analyses

Venous blood (~5 mL) was collected from the antecubital vein after an overnight fast (10–12 h) into EDTA and plain Vacutainer tubes by trained phlebotomists. EDTA-anticoagulated whole blood was promptly analyzed for complete blood count (CBC) on an automated hematology analyzer (ABX Micros ES 60, HORIBA Medical, France) (43). Calibration was performed using the manufacturer's ABX Minocal calibrator, and quality control was monitored by ABX Minotrol 16 whole-blood controls (low, normal, and high levels). Serum was separated from blood collected in plain tubes *via* centrifugation at 3,000 rpm for 10 min. Aliquots were stored at −80 C until further biochemical analyses. Serum concentrations of high-sensitivity C-reactive protein (hs-CRP, mg/L) and alpha-1-acid glycoprotein 2 (AAGP2, g/dL) were determined using an automated chemistry analyzer (cobas^®^ c 311, Roche Diagnostics, Germany).

### 2.7 Dietary intake

Dietary intake was assessed using three non-consecutive 24-h dietary recalls. For athletes, recalls were conducted for one heavy training day, one moderate training day, and one rest or low-intensity day; for controls, two working days and one non-working day were included. All interviews were conducted by trained nutritionists utilizing a standardized portion-size estimation aid (12). Dietary recalls from athletes were rigorously cross-validated against contemporaneous food diaries. Nutrient intakes were analyzed using NSR-Nutrical software, referencing the Indian Food Composition Tables (13) and the USDA database (2019), to quantify energy, macro- and micronutrients, fatty acids, and dietary fiber. The Dietary Inflammatory Index (DII) was calculated as described by Shivappa et al. (14).

### 2.8 Fecal sample collection and quantitative qPCR analysis

Fecal samples were collected in sterile containers, immediately transported on dry ice, and stored at −80 C until analysis. Genomic DNA was isolated from approximately 220–250 mg of stool using the QIAGEN extraction kit (QIAGEN, Germany) with an additional mechanical lysis step, as described by Lim et al. (15). DNA concentration and purity were assessed using a Multiskan SkyHigh microplate spectrophotometer (Thermo Fisher Scientific) by measuring absorbance at 260 nm and protein contamination at 230 nm. Quantification of target bacterial taxa was performed *via* SYBR Green-based quantitative real-time PCR (qPCR), focusing on taxa implicated in athletic performance, inflammation, and energy metabolism. PCR reactions (20 μL) contained 10 μL Takyon™ Low ROX SYBR MasterMix (Eurogentec, Belgium), 0.4 μM each of forward and reverse primers, 2.5 μL of extracted Fecal DNA, and nuclease-free water. Primer sequences, target specificity, and relevant references are detailed in Supplementary Table 1. Amplification was conducted on a LightCycler^®^ 480 II system (Roche Diagnostics, Switzerland), with an initial denaturation at 95°C for 10 min, followed by 40 cycles of 95°C for 10 s and 60°C for 60 s. Annealing temperatures were optimized for each primer pair (58–62°C), with final conditions provided in Supplementary Table 1. Specificity of amplification was verified by melt-curve analysis. Negative (no-template) controls were included in each run, and all assays were performed in technical duplicate. Universal bacterial 16S rRNA gene amplification served as the internal reference for normalization. Relative abundances of target taxa were calculated using the 2^−^ΔCt method, where ΔCt is the difference between the Ct value of the target and that of universal 16S rRNA. Results are reported as relative abundance within the total bacterial DNA pool. For statistical analysis, non-transformed 2^−^ΔCt values were used, while log-transformed values were applied for data visualization.

### 2.9 Statistics

Anthropometric data, dietary intake, and stool specimens were collected from all participants. Due to logistical constraints, data availability varied across specific assessments: resting metabolic rate (RMR) was obtained from 27 endurance athletes, 25 strength athletes, and 18 sedentary controls; blood parameters from 26 endurance athletes, 21 strength athletes, and 18 controls; and DEXA measurements from 25 endurance athletes, 13 strength athletes, and 18 controls. Missing data, particularly DEXA among strength athletes, were primarily due to participants relocating for specialized pre-competition training. Analyses were conducted using all available data, and no imputation was performed.

Statistical analyses and data visualization were performed using GraphPad Prism (version 8.0.2). Data distribution was assessed for normality using the Shapiro-Wilk test. Normally distributed variables are presented as mean ± standard deviation (SD) and were compared across groups using one-way analysis of variance (ANOVA) followed by Tukey's *post hoc* test. Non-normally distributed variables are presented as medians (interquartile ranges) and were analyzed using the Kruskal–Wallis test, with Dunn's *post hoc* test for multiple comparisons. A two-sided *p*-value < 0.05 was considered statistically significant. Associations between dietary intake variables and the selected gut microbial taxa were evaluated using Spearman's rank correlation coefficient (ρ). Effect sizes were also calculated to quantify the magnitude of between-group differences. This aided interpretation beyond statistical significance, particularly in the context of unequal sample sizes. Effect sizes were expressed as eta-squared (η^2^) and interpreted as trivial (< 0.01), small (0.01–0.06), moderate (0.06–0.14), or large (>0.14).

## 3 Results

### 3.1 . Participant characteristics and body composition

Participant characteristics and DEXA-derived body composition metrics are summarized in Table 1. No significant differences in age or height were observed between groups (*p* > 0.17). Strength athletes had significantly higher body mass and BMI than both endurance athletes and controls (*p* < 0.01). Distinct body composition phenotypes were evident by training modality. Endurance athletes displayed the lowest body fat percentage, whereas strength athletes exhibited greater body mass, lean body mass, fat-free mass, trunk lean mass, and appendicular skeletal muscle mass relative to both comparison groups (all *p* < 0.01). Regional analysis demonstrated that endurance athletes possessed significantly lower arm and leg fat mass compared to controls (see Supplementary Table 2), while strength athletes consistently showed the highest absolute lean mass across all anatomical regions.

**Table 1:** Participant characteristics and body composition details.

Variable	Sedentary controls (*n* = 24)	Endurance athletes (*n* = 27)	Strength athletes (*n* = 25)	*p*-value
Age (years)	25 ± 2	26 ± 4	25 ± 4	0.224
Height (cm)	171.2 ± 6.0	172.1 ± 4.8	174.4 ± 8.1	0.172
Body mass (kg)	64.40 ± 7.46^†^	64.29 ± 8.70	83.10 ± 21.74^‡^	0.002
BMI (kg/m^2^)	22.0 ± 2.6^†^	21.6 ± 2.1	26.9 ± 5.5^‡^	0.001
Body composition				
Body fat percentage (%)	24.28 ± 6.31^*^	19.70 ± 5.91	21.32 ± 5.06	0.030
Lean body mass (kg)	46.46 ± 4.29^†^	48.17 ± 5.38	56.80 ± 12.25^‡^	0.001
Fat mass (kg)	15.97 ± 5.32	12.75 ± 5.16	16.67 ± 6.40	0.037
Fat-free mass (kg)	48.78 ± 4.50^†^	50.76 ± 5.74	59.59 ± 12.62^‡^	0.001
ASM (kg)	21.41 ± 1.88^†^	22.45 ± 2.52	26.44 ± 5.78^‡^	0.001
ASM/height (kg/m^2^)	0.12 ± 0.01^†^	0.13 ± 0.01	0.15 ± 0.03^‡^	0.000
Total body BMD (g/cm^2^)	1.12 ± 0.09^*^^†^	1.21 ± 0.12	1.25 ± 0.13	0.005

Data are presented as mean ± SD. Group differences were assessed using one-way analysis of variance (ANOVA). Effect sizes are reported as eta squared (η^2^) and interpreted as small (η^2^ = 0.01–0.05), moderate (η^2^ = 0.06–0.13), or large (η^2^ ≥ 0.14). Symbols indicate.

*Significant difference between Control and Endurance groups (*p* < 0.05).

†Significant difference between Control and Strength groups (*p* < 0.05).

‡Significant difference between Endurance and Strength groups (*p* < 0.05).

### 3.2 Bone mineral content and bone mineral density

Total bone mineral content (BMC) varied significantly between groups (*p* = 0.013), with sedentary controls demonstrating lower BMC (2,323.48 ± 308.83 g) than both endurance (2,582.70 ± 450.81 g) and strength athletes (2,793.42 ± 504.99 g). Total bone mineral density (BMD) also differed significantly across groups (*p* = 0.005), with controls exhibiting the lowest BMD (1.12 ± 0.09 g/cm^2^), followed by endurance (1.21 ± 0.12 g/cm^2^) and strength athletes (1.25 ± 0.13 g/cm^2^). Strength athletes consistently showed higher BMD and BMC than both endurance athletes and controls across most skeletal sites, including the lumbar spine, ribs, pelvis, and subtotal regions (all *p* ≤ 0.01). Endurance athletes had significantly greater leg BMD than controls (*p* = 0.0003), whereas leg BMC was higher in strength athletes than in controls (*p* < 0.005). No significant differences were observed among groups for head BMD or BMC. Comprehensive site-specific BMC and BMD data are available in Supplementary Table 3.

### 3.3 Resting metabolic rate and substrate utilization

**Figure 2:**
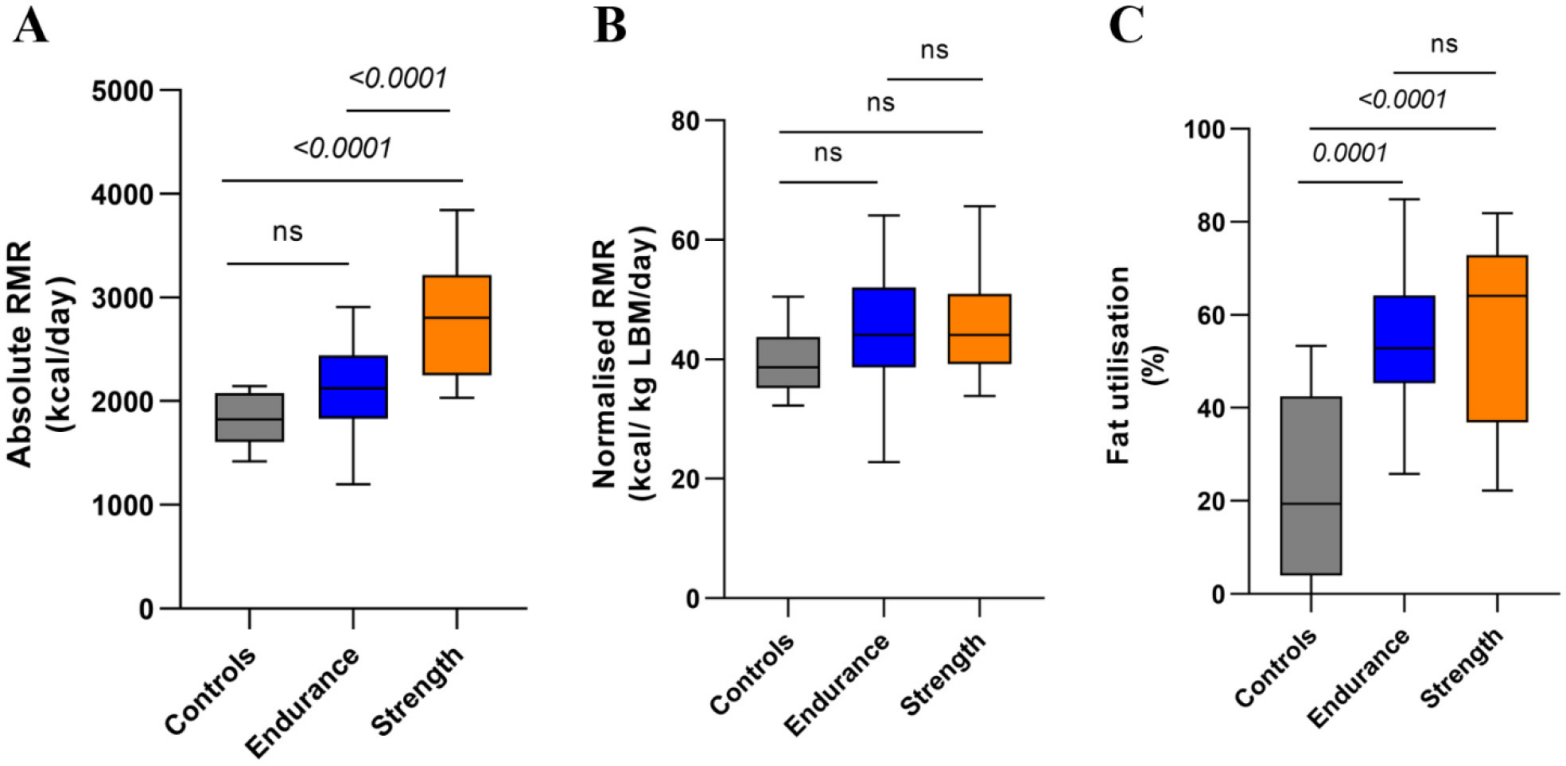
Resting metabolic rate and fasting fat utilization among sedentary controls, endurance athletes, and strength athletes. **(A)** Absolute resting metabolic rate (RMR; kcal/day), **(B)** RMR normalized to lean body mass (kcal/kg LBM/day), and **(C)** fasting fat utilization (%). Statistical significance between groups is indicated above the plots (*p* < 0.05), while ns denotes non-significant differences.

Absolute resting metabolic rate (RMR) differed significantly across groups (*p* < 0.001), with strength athletes exhibiting the highest RMR (2,215 ± 412 kcal/day), followed by endurance athletes (1,987 ± 356 kcal/day), and controls (1,624 ± 298 kcal/day; Figure 2). When normalized to lean body mass, RMR did not differ significantly between groups (controls: 40 ± 6 kcal/kg LBM/day; endurance athletes: 45 ± 9 kcal/kg LBM/day; strength athletes: 46 ± 9 kcal/kg LBM/day; *p* = 0.09). Fasting substrate utilization, as assessed by respiratory quotient (RQ), also varied significantly among groups. Endurance athletes demonstrated the lowest RQ (0.79 ± 0.05), followed by strength athletes (0.81 ± 0.06), with controls exhibiting the highest RQ (0.88 ± 0.06), indicating a greater reliance on fat oxidation in both athlete groups relative to controls (*p* < 0.0001).

### 3.4 Hematological parameters

**Figure 3:**
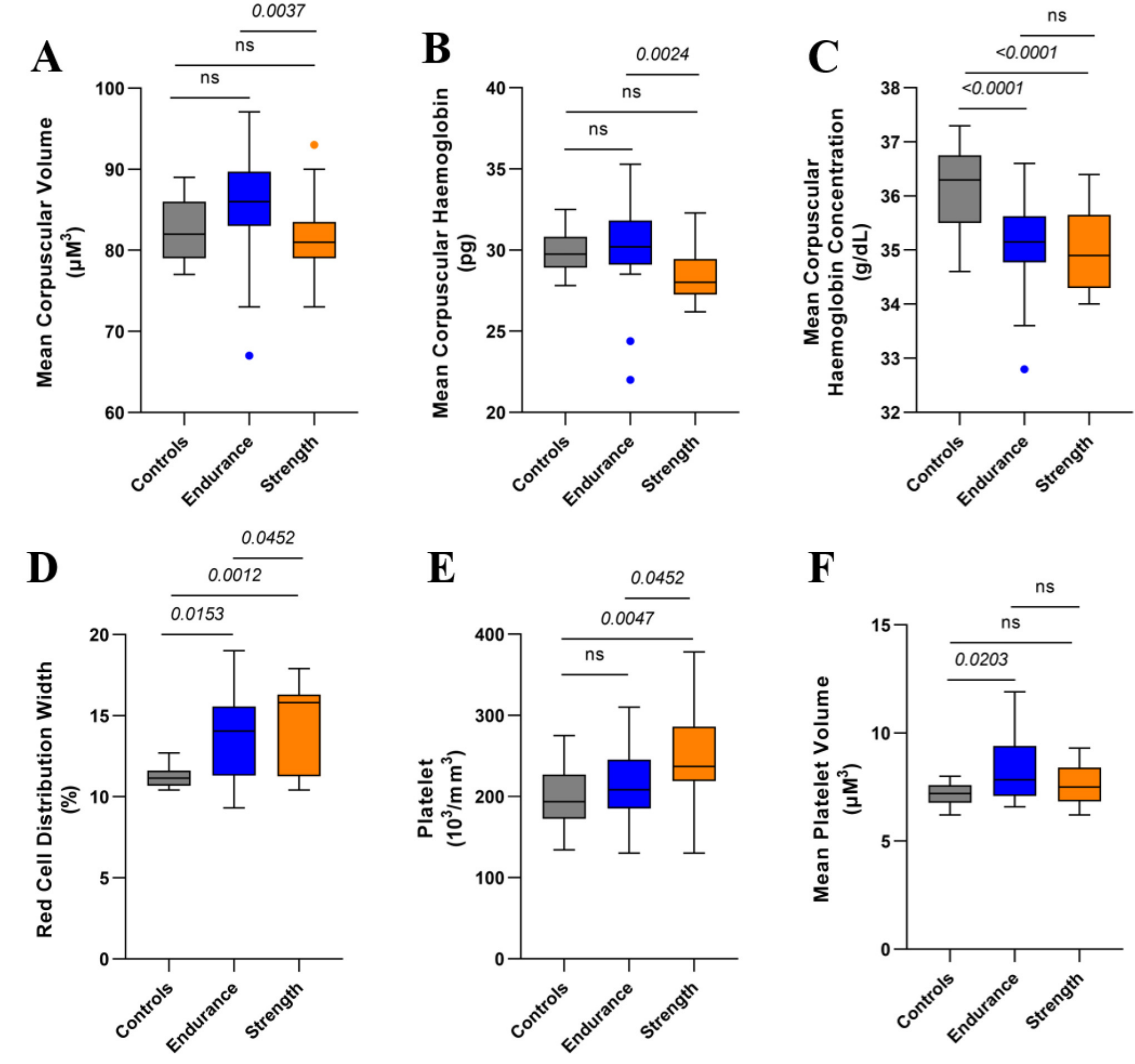
Box plots showing group differences in hematological indices: **(A)** Mean Corpuscular Volume, **(B)** Mean Corpuscular Hemoglobin, **(C)** Mean Corpuscular Hemoglobin Concentration, **(D)** Red Cell Distribution Width, and **(E)** Platelet **(F)** Mean Platelet Volume across groups. Overall group effects were significant for all parameters (*p* < 0.05). Boxes represent IQR, horizontal lines indicate medians, and whiskers denote IQR.

Significant group differences were observed in selected hematological parameters (Figure 3). Mean corpuscular volume (MCV) and mean corpuscular hemoglobin (MCH) were significantly higher in endurance athletes compared to strength athletes (*p* < 0.05). Mean corpuscular hemoglobin concentration (MCHC) was highest in controls, intermediate in endurance athletes, and lowest in strength athletes (overall *p* < 0.0001). Coefficient of variation of red cell distribution width (RDW-CV) was elevated in both athlete groups relative to controls. Platelet count was significantly greater in strength athletes than in controls (*p* = 0.0047), while mean platelet volume was higher in endurance athletes than in controls (*p* = 0.0247). No significant group differences were detected for hemoglobin concentration, hematocrit, white blood cell count, plateletcrit, platelet distribution width, or granulocyte-to-lymphocyte ratio.

### 3.5 Dietary intake

**Figure 4:**
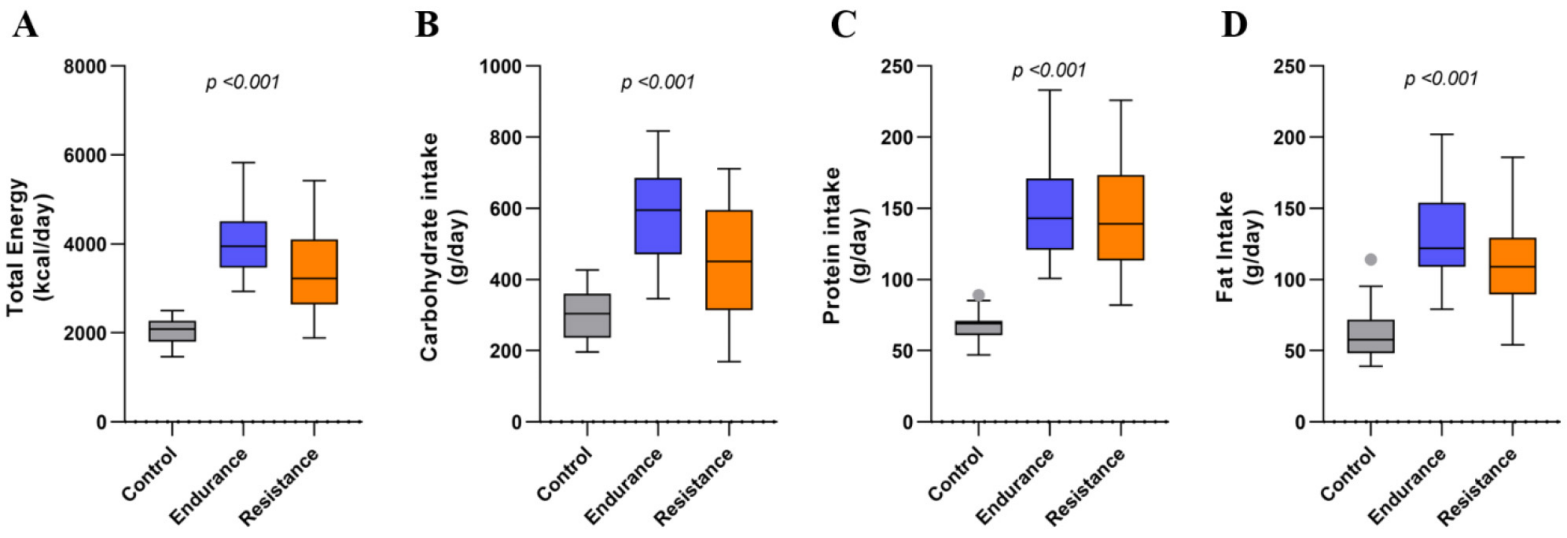
Differences in dietary intake between sedentary controls, endurance and strength athletes. **(A)** Total energy, **(B)** Carbohydrate intake, **(C)** Protein intake, and **(D)** Fat intake. Overall group effects were significant for all parameters (*p* < 0.001). Boxes represent IQR, horizontal lines indicate medians, and whiskers denote IQR. Dots refer to outliers.

Dietary intake differed significantly between groups (Table 2). Endurance athletes reported the highest energy intake (4,074 ± 742 kcal/day), followed by strength athletes (3,406 ± 871 kcal/day) and healthy controls (2,035 ± 326 kcal/day; *p* < 0.0001) (Figure 4). Similarly, endurance and strength athletes consumed significantly greater amounts of carbohydrate, protein, fat, and dietary fiber than controls (all *p* < 0.0001).

**Table 2:** Differences in nutrient intake in participants.

Nutrient intake variable	Sedentary controls (*n* = 24)			Endurance athletes (*n* = 27)			Strength athletes (*n* = 25)			*p-value*	η^2^
	Mea*n* ±SD	Median	IQR	Mea*n* ±SD	Median	IQR	Mea*n* ±SD	Median	IQR		
Energy (kcal)	2,035 ± 326^*^^†^	2,083	354	4,074 ± 742	3,947	1,025	3,406 ± 871^‡^	3,232	1,403	< 0.0001	0.152
Carbohydrate (g)	303 ± 75^*^^†^	304	117	576 ± 121	595	203	449 ± 155^‡^	451	270	< 0.0001	0.156
Protein (g)	67 ± 11^*^^†^	69	8	150 ± 35	143	48	146 ± 40	139	59	< 0.0001	0.242
Fat (g)	62 ± 19^*^^†^	57	19	130 ± 31	122	41	114 ± 31	109	33	< 0.0001	0.478
Total mono unsaturated fatty acids (TMUFA) (g)	25.2 ± 8.3^*^^†^	23.3	6	45.3 ± 10.6	46.2	11.3	40.0 ± 12.0	40	15.1	< 0.0001	0.353
Total polyunsaturated fatty acids (TPUFA) (g)	14.2 ± 4.1^*^^†^	12.9	3.4	28.5 ± 9.4	28.9	8.3	23.2 ± 7.0^‡^	23.9	9.5	< 0.0001	0.414
Total saturated fatty acids (TSFA) (g)	16.7 ± 7.4^*^^†^	14.1	6.7	41.4 ± 13.8	40.3	17.5	34.5 ± 12.9	29.9	19.8	< 0.0001	0.425
Total dietary fiber (g)	36 ± 9^*^^†^	35	15	79 ± 23	79	30	65 ± 27^‡^	64	32	< 0.0001	0.153
Insoluble dietary fiber (g)	28 ± 7^*^^†^	26	12	56 ± 16	58	19	48 ± 20	48	25	< 0.0001	0.144
Soluble dietary fiber (g)	8 ± 2^*^^†^	8	2	17 ± 5	17	6	14 ± 6^‡^	13	8	< 0.0001	0.198
Total energy from carbohydrate (%)	59.9 ± 7.2^†^	59.9	11.6	56.5 ± 5.3	56.9	6.2	51.8 ± 8.9	52.3	14.2	0.007	0.112
Total energy from protein (%)	13.2 ± 1.3^*^^†^	13.2	2.2	14.6 ± 1.1	14.6	1	17.4 ± 2.9^‡^	16.7	2.8	< 0.0001	0.494
Total energy from total fat (%)	26.9 ± 7.0	26.9	10	28.9 ± 5.8	28.1	6.3	30.9 ± 7.1	31.3	11.6	0.129	0.029

Data are presented as mean ± standard deviation (SD), median, and interquartile range (IQR). Group differences were assessed using one-way analysis of variance (ANOVA) for normally distributed variables. *Post-hoc* pairwise comparisons were performed using Bonferroni correction. Effect size is reported as eta-squared (η^2^).

*Significant difference between Control and Endurance groups (*p* < 0.05).

†Significant difference between Control and Strength groups (*p* < 0.05).

‡Significant difference between Endurance and Strength groups (*p* < 0.05).

Endurance athletes reported the highest absolute carbohydrate intake (576 ± 121 g/day), whereas strength athletes derived a greater proportion of total energy from protein (17.4 ± 2.9%) compared with endurance athletes (14.6 ± 1.1%) and controls (13.2 ± 1.3%; *p* < 0.0001). Total dietary fiber intake was also significantly higher in endurance (79 ± 23 g/day) and strength athletes (65 ± 27 g/day) than in controls (36 ± 9 g/day; *p* < 0.0001).

Significant differences were also observed in dietary fatty acid intake. Endurance and strength athletes consumed higher amounts of monounsaturated, polyunsaturated, and saturated fatty acids than controls (all *p* < 0.0001). In addition, micronutrient intake differed substantially among groups (Supplementary Table 4). Both athlete groups reported significantly greater intakes of several vitamins and minerals, including vitamins A, D, E, K, B-complex vitamins, vitamin C, iron, magnesium, selenium, and zinc, compared with healthy controls (all *p* < 0.05). Athletes also consumed higher amounts of linoleic acid and α-linolenic acid than controls (*p* < 0.0001), while docosahexaenoic acid intake was highest among strength athletes (*p* = 0.008).

Dietary Inflammatory Index (DII) scores differed significantly by group (controls: 0.003; endurance athletes: −3.258; strength athletes: −2.784; *p* < 0.001), indicating a more anti-inflammatory dietary pattern in athletes. Total dietary fiber intake showed weak positive correlations with the relative abundance of *Prevotella copri* (ρ = 0.263, *p* = 0.012) and *Methanobrevibacter smithii* (ρ = 0.257, *p* = 0.027), and moderate negative correlations with *Lactobacillus* (ρ = −0.432, *p* < 0.001) and Enterobacteriaceae (ρ = −0.387, *p* < 0.001). Additional associations between soluble and insoluble dietary fiber intake and microbial relative abundance are presented in Supplementary Table 5.

### 3.6 Gut microbial signatures

**Figure 5:**
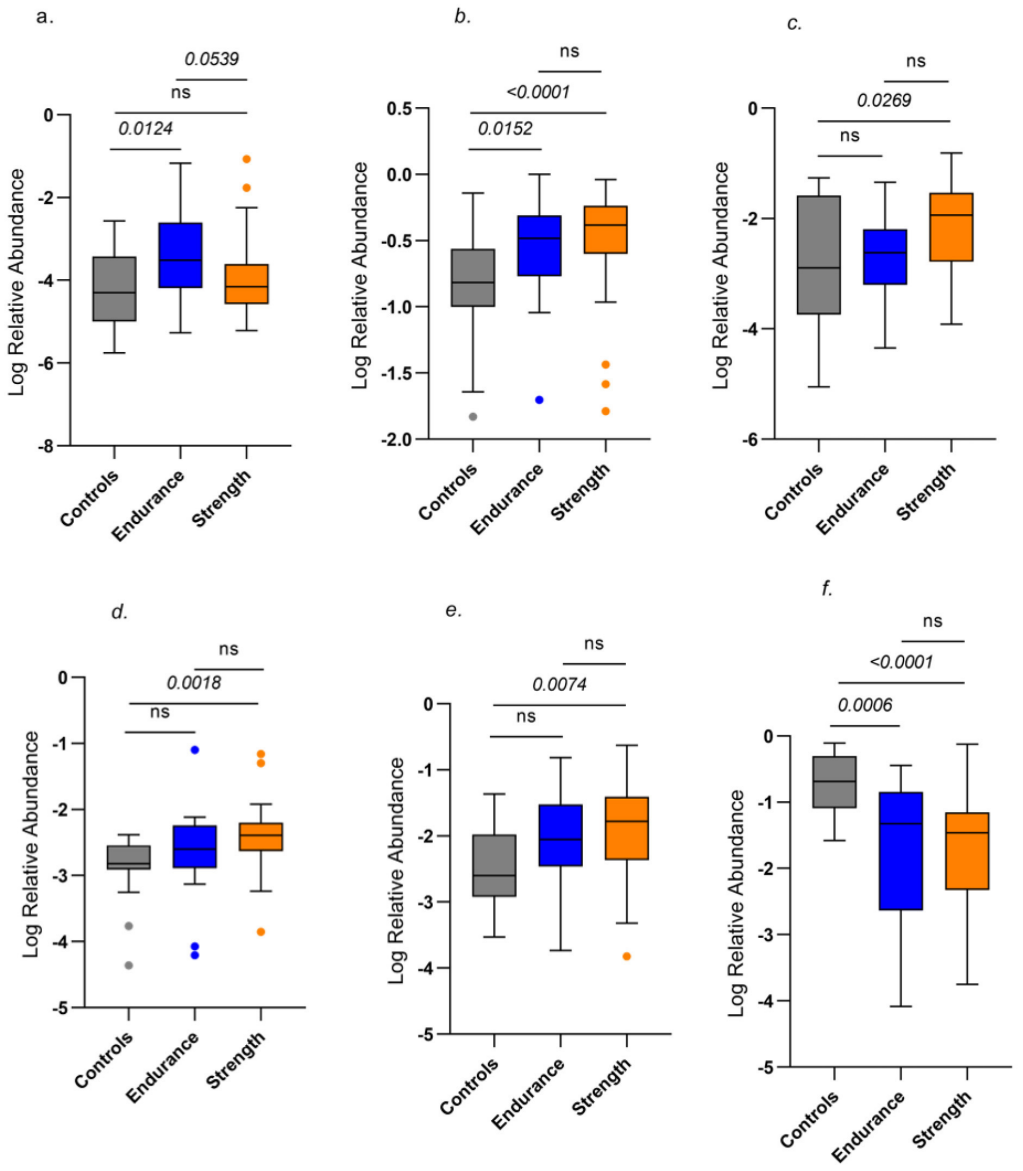
Box plots illustrating group-wise differences in the relative abundance of **(a)** *Methanobrevibacter smithii*, **(b)** *Bacteroides* spp., **(c)** *Prevotella copri*, **(d)** *Desulphovibrio* spp., **(e)** *Streptococcus* spp., and **(f)** *Lactobacillus* spp. Boxes represent IQR, horizontal lines indicate medians, and whiskers denote IQR. Dots represent outliers.

The relative abundance of selected gut microbial markers differed significantly across groups (Figure 5). *Bacteroides* abundance also demonstrated a large effect (η^2^ = 0.223, L), with the highest levels observed in strength athletes, followed by endurance athletes and controls (*p* < 0.0001). *Prevotella copri* showed a small but significant group difference (η^2^ = 0.058, S), with higher levels in strength athletes than in controls (*p* = 0.0269). *Desulphovibrio* abundance was elevated in strength athletes relative to controls (η^2^ = 0.148, L; *p* = 0.001), while *Streptococcus* abundance was also higher in strength athletes (η^2^ = 0.181, L; *p* < 0.001). *Methanobrevibacter smithii* abundance was higher in endurance athletes than in controls, with a moderate effect size (η^2^ = 0.087, M; *p* = 0.0214). *Lactobacillus* spp. abundance showed a large group effect (η^2^ = 0.239, L), with the highest levels in controls, intermediate levels in endurance athletes, and the lowest in strength athletes (*p* < 0.0001). No significant group differences were observed for *Bifidobacterium* (η^2^ = 0.007, Triv), *Akkermansia muciniphila* (η^2^ = 0.018, S), and *Enterobacteriaceae* (η^2^ = 0.012, S). A moderate negative correlation was observed between *Lactobacillus* spp. relative abundance and total bone mineral density (Spearman *r* = −0.46, *p* = 0.0003).

## 4 Discussion

This study demonstrates that elite Indian endurance and strength athletes exhibit clear training-modality–specific physiological phenotypes across body composition, bone health, resting metabolism, hematological indices, and dietary intake. Despite these robust sport-specific adaptations, absolute lean mass and bone indices in both athlete groups remained lower than values commonly reported in non-Indian elite cohorts (16–19). This may reflect population-specific morphological characteristics that could influence the absolute expression of training-induced adaptations.

### 4.1 Differences in body composition

Body composition is a critical determinant of athletic performance, metabolic health, and injury risk. Ethnic variability in adiposity, lean mass, and skeletal characteristics poses significant challenges for direct comparisons across athletic populations (16, 20). Prior research indicates that Indian adults, in particular, tend to have a greater proportion of adiposity and reduced lean mass and bone mineral content relative to several other ethnic groups at equivalent BMI values (6). Within this context, the sport-specific differences observed in our cohort largely mirror established trends in endurance and strength athletes. Our data showed that endurance athletes had lower adiposity than strength athletes, while the latter had greater lean mass, appendicular skeletal muscle mass, and bone density. Nevertheless, the absolute measures of lean mass and bone indices among our participants were generally lower than those reported in Western athletic cohorts (17, 18, 21). Collectively, these findings underscore the influence of population-specific factors on body composition, while reaffirming the expected physiological adaptations associated with endurance and strength training.

### 4.2 Differences in physiology

The higher absolute resting metabolic rate (RMR) observed in strength athletes compared with endurance athletes and controls closely paralleled differences in fat-free mass, reflecting the established association between lean tissue and resting energy expenditure. RMR values in endurance athletes were comparable to those reported in other trained populations, while the elevated RMR in strength athletes aligns with prior evidence linking greater muscle mass to increased resting energy expenditure (19, 22). Although RMR normalized to lean body mass did not differ significantly between groups, athletes tended to exhibit slightly higher values than controls, possibly reflecting long-term training adaptations.

Fasting substrate utilization further distinguished athletes from controls. Both endurance and strength athletes exhibited lower fasting respiratory quotient values than sedentary controls, consistent with a greater reliance on fat oxidation at rest. This finding is consistent with previous reports in endurance-trained cohorts and may be consistent with enhanced metabolic flexibility due to regular training (23).

### 4.3 Differences in hematology parameters

Endurance athletes displayed elevated mean corpuscular volume (MCV) and mean corpuscular hemoglobin (MCH) relative to strength athletes, which may reflect distinct erythrocyte characteristics associated with chronic aerobic training (24). These findings corroborate prior research in endurance-trained cohorts, where such hematological characteristics have been associated with physiological adaptations from sustained aerobic loading (25). Conversely, the absence of a similar increase in MCV among strength athletes highlights possible modality-specific differences in hematological responses.

Both athlete groups exhibited greater red cell distribution width (RDW-CV) than controls, with the highest values found in strength athletes, reflecting increased heterogeneity within erythrocyte populations. This observation aligns with previous reports of altered erythrocyte turnover among trained athletes. Platelet count was modestly elevated in strength athletes compared to controls, though all values remained within clinical reference ranges; this may be related to repeated mechanical loading and tissue remodeling (26, 27). Additionally, mean platelet volume was higher in endurance athletes than in controls, consistent with findings from other endurance-trained cohorts (28). No significant differences in basal inflammatory markers were observed, indicating a similar inflammatory status across all groups at the time of measurement.

### 4.4 Differences in nutrient intake

Dietary intake patterns differed substantially between athletes and sedentary controls and were generally consistent with the metabolic demands of their respective training regimens. Endurance athletes reported the highest habitual energy intake, followed by strength athletes and controls, suggesting that energy intake was broadly aligned with training demands during the general preparation phase. This contrasts with reports of low energy availability in some athletic populations (29–31). Recent evidence has further highlighted the importance of adequate energy availability for metabolic regulation, perception of effort, and endurance performance (32). Notably, the present data does not support presence of chronic under-fuelling among endurance athletes.

Macronutrient intake also reflected differences in training demands. Protein intake increased progressively from controls to endurance athletes and strength athletes, consistent with established sports nutrition recommendations. While endurance athletes reported a lower proportional contribution of carbohydrate to total energy intake than controls, their higher overall energy consumption resulted in greater absolute carbohydrate intake, which may be more relevant for supporting training and recovery.

Beyond total energy and macronutrient intake, dietary quality differed between groups. Endurance athletes reported higher intakes of monounsaturated and polyunsaturated fatty acids, dietary fiber, and several micronutrients, accompanied by lower Dietary Inflammatory Index scores compared with controls. These findings emphasize the importance of evaluating dietary quality alongside energy and macronutrient intake when assessing nutritional adequacy in athletic populations.

Importantly, the substantial differences in energy intake, macronutrient distribution, fiber consumption, and fatty acid intake observed between groups should be considered when interpreting the gut microbiota findings. Given the well-established influence of diet on microbial composition, these dietary factors may have contributed to the observed differences in bacterial taxa and therefore represent potential confounders in the relationship between training status and gut microbial profiles.

### 4.5 Differences in gut bacterial taxa

Both endurance and strength athletes exhibited a greater relative abundance of *Bacteroides* spp., *Prevotella copri, Desulphovibrio* spp., and *Streptococcus* spp., with endurance athletes exhibiting a higher relative abundance of *Methanobrevibacter smithii*. The pronounced differences in dietary intake between groups, particularly in total energy, protein, fiber, and fatty acids, likely may have contributed to the observed microbial differences. Consequently, the independent effects of training modality and dietary intake cannot be disentangled within the confines of this cross-sectional design. Nevertheless, the gut microbial profiles identified in athletes are largely consistent with patterns previously described in other physically active populations.

The enrichment of *Bacteroides* spp. and *Desulphovibrio* spp. observed in athletes are consistent dietary differences, particularly higher protein and fat intake, in accordance with findings from other athletic cohorts (33, 34). Similarly, the elevated abundance of *Prevotella copri* in athletes is consistent with previous research reporting associations between this taxon, carbohydrate-rich diets, and prolonged training exposure (35). The greater prevalence of *Methanobrevibacter* smithii in endurance athletes also aligns with earlier reports from endurance-trained populations (8); however, the functional implications of this finding remain indeterminate based on current data.

*Lactobacillus* spp. abundance was higher in controls than in athletes. Although *Lactobacillus* is traditionally regarded as beneficial, emerging evidence indicates that its abundance is not universally linked to favorable metabolic or musculoskeletal health across populations (36). The comparatively lower levels in athletes are consistent with reports documenting increased *Lactobacillus* abundance among sedentary or higher-adiposity groups (37–40). Of note, a moderate inverse association emerged between *Lactobacillus* abundance and bone mineral density; however, the mechanistic basis for this relationship remains unclear. The elevated *Lactobacillus* levels in less-active controls, coupled with their reduced bone mineral density, underscore the need for further research into the interplay between the gut microbiota and skeletal health.

Given that this study evaluated only the relative abundance of selected taxa through targeted qPCR, direct functional or metabolic inferences cannot be drawn and must be substantiated by future metagenomic, metabolomic, and mechanistic investigations. Prior studies from South Korea and Ireland have identified minimal differences in overall microbial diversity between endurance and strength athletes (41, 42). Although those investigations used broader sequencing methodologies, the targeted qPCR approach employed here allowed focused quantification of key microbial taxa.

### 4.6 Strengths, limitations, and applied relevance

The cross-sectional design of this study precludes definitive conclusions regarding temporal relationships or causality among training modality, dietary intake, physiological characteristics, and gut microbial composition. Differences in recruitment environments between athletes and controls may have introduced selection bias or unmeasured confounding factors that could have influenced the observed associations. As an exploratory study in elite athletes, a formal *a priori* sample size calculation was not performed, and unequal data availability across assessments resulted in smaller subsamples for certain outcomes, particularly DEXA measurements in strength athletes. This may have reduced statistical power and increased uncertainty around some body composition and bone density estimates. Dietary intake was evaluated using self-reported 24-h recalls, which may introduce recall bias and misreporting, despite cross-verification with food diaries. Gut microbiota profiling utilized targeted quantitative PCR (qPCR), allowing sensitive quantification of selected taxa but lacking comprehensive insight into microbial diversity, function, or metabolic pathways. Although participants abstained from structured exercise for at least 12 h prior to resting metabolic rate assessment, residual acute effects of recent training cannot be fully excluded. Finally, as the cohort consisted solely of male athletes, the generalizability of these findings to female athletic populations is inherently limited.

Despite these limitations, this study utilized gold-standard methodologies and provides an integrated physiological, nutritional, and microbial characterization of elite endurance and strength athletes during a standardized training phase. The findings yield nuanced, contextually relevant insights into elite Indian athletes and offer a foundation for future research and interpretation of training-induced adaptations.

## 5 Conclusions

In conclusion, endurance and strength athletes displayed distinct profiles in body composition, bone health, dietary intake, hematological indices, and gut microbiota relative to sedentary controls. While resting metabolic rate normalized to lean body mass and fasting substrate utilization did not differ significantly between the athlete groups, both athletic cohorts exhibited pronounced physiological differences compared to non-athletes. These findings enhance our understanding of sport modality–specific physiological and microbial adaptations in elite Indian athletes and provide a foundation for future research and the development of tailored nutrition and training strategies. Expanding future studies to include athletes from additional categories within Mitchell's classification, particularly those involved in sports characterized by both high dynamic and high static demands, will further elucidate the spectrum of gut microbial and physiological diversity across athletic populations.

## Data Availability

The original contributions presented in the study are included in the article/Supplementary material, further inquiries can be directed to the corresponding author.
