# Supplementary material for: Training modality-specific differences in body composition, resting metabolic rate, diet, and gut microbial signatures in elite endurance and strength athletes
Source: Front Nutr. 2026 Jul 13;13:1844692. doi: 10.3389/fnut.2026.1844692 (PMC13402902; doi:10.3389/fnut.2026.1844692)
Supplement: Supplementary file 1 [file Supplementary_file_1.docx]

Supplementary Material

**Supplementary Table 1.** RT-PCR primer sequences

| **Organism** | **Forward/Reverse** | **Sequence** | **Source Article** | **DNA Template conc. Used in each reaction** |
| --- | --- | --- | --- | --- |
| *Lactobacillus spp.* | Forward | 5’- AGCAGTAGGGAATCTTCCA-3' | [1] | 0.1ng/ 2.5 µL |
|  | Reverse | 5’-CGCCACTGGTGTTCYTCCATATA- 3’ | [1] | 0.1ng/ 2.5 µL |
| *Fecalibacterium* | Forward | 5’-GATGGCCTCGCGTCCGATTAG-3’ | [2] | 0.1ng/ 2.5 µL |
|  | Reverse | 5’-CCGAAGACCTTCTTCCTCC-3’ | [2] | 0.1ng/ 2.5 µL |
| *Enterobacteriaceae* | Forward | 5’-CATTGACGTTACCCGCAGAAGAAGC-3’ | [3] | 100 ng/ 2.5 µL |
|  | Reverse | 5’-CTCTACGAGACTCAAGCTTGC-3’ | [3] | 100 ng/ 2.5 µL |
| *Bifidobacterium spp.* | Forward | 5’- ATCTTCGGACCBGAYGAGAC-3’ | [4] | 100 ng/ 2.5 µL |
|  | Reverse | 5’-CGATVACGTGVACGAAGGAC-3’ | [4] | 100 ng/ 2.5 µL |
| *Bacteroides spp.* | Forward | 5’-GAAGGTCCCCCACATTG-3’ | [5] | 0.1ng/ 2.5 µL |
|  | Reverse | 5’-CGCKACTTGGCTGGTTCAG-3’ | [5] | 0.1ng/ 2.5 µL |
| *Total bacteria (universal 16S rRNA)* | Forward | 5’-ACTCCTACGGGAGGCAGCAG-3’ | [6] | 0.1ng/ 2.5 µL |
|  | Reverse | 5’-ATTACCGCGGCTGCTGG-3’ | [6] | 0.1ng/ 2.5 µL |
| *Akkermansia muciniphila* | Forward | 5’-CAGCACGTGAAGGTGGGGAC-3’ | [7] | 50 ng/ 2.5 µL |
|  | Reverse | 5’-CCTTGCGGTTGGCTTCAGAT-3’ | [7] | 50 ng/ 2.5 µL |
| *Methanobrevibacter smithii* | Forward | 5’-CCGGGTATCTAATCCGGTTC-3’ | [8] | 100 ng/ 2.5 µL |
|  | Reverse | 5’-CTCCCAGGGTAGAGGTGAAA-3’ | [8] | 100 ng/ 2.5 µL |
| *Desulphovibrio* | Forward | 5’-CCGTAGATATCTGGAGGAACATCAG-3’ | [9] | 100 ng/ 2.5 µL |
|  | Reverse | 5’-ACATCTAGCATCCATCGTTTACAGC-3’ | [9] | 100 ng/ 2.5 µL |
| *Prevotella copri* | Forward | 5’-GAGACGGGCTGGAGTGATTT-3’ | [10] | 10 ng/ 2.5 µL |
|  | Reverse | 5’-TCCGTGTGGCTTTCATGGAG-3’ | [10] | 10 ng/ 2.5 µL |
| *Streptococcus genus* | Forward | 5’-GTACAGTTGCTTCAGGACGTATC-3’ | [11] | 10 ng/ 2.5 µL |
|  | Reverse | 5’-ACGTTCGATTTCATCACGTTG-3’ | [11] | 10 ng/ 2.5 µL |

All primer pairs were standardized in-house prior to analysis. Specificity was confirmed by melt-curve analysis, and annealing temperatures were optimized for each target taxon. Reaction conditions were maintained constant across samples following optimization**.**

**References:**

[1] J.-P. Furet *et al.*, “Comparative assessment of human and farm animal faecal microbiota using real-time quantitative PCR: Human and farm animal faecal microbiota,” *FEMS Microbiology Ecology*, vol. 68, no. 3, pp. 351–362, Jun. 2009, doi: 10.1111/j.1574-6941.2009.00671.x.

[2] Q. Gui *et al.*, “The association between gut butyrate-producing bacteria and non-small-cell lung cancer,” *J Clin Lab Anal*, vol. 34, no. 8, p. e23318, Aug. 2020, doi: 10.1002/jcla.23318.

[3] D. N. Frank, A. L. St. Amand, R. A. Feldman, E. C. Boedeker, N. Harpaz, and N. R. Pace, “Molecular-phylogenetic characterization of microbial community imbalances in human inflammatory bowel diseases,” *Proc. Natl. Acad. Sci. U.S.A.*, vol. 104, no. 34, pp. 13780–13785, Aug. 2007, doi: 10.1073/pnas.0706625104.

[4] C. Ramirez-Farias, K. Slezak, Z. Fuller, A. Duncan, G. Holtrop, and P. Louis, “Effect of inulin on the human gut microbiota: stimulation of *Bifidobacterium adolescentis* and *Faecalibacterium prausnitzii*,” *Br J Nutr*, vol. 101, no. 4, pp. 541–550, Jul. 2008, doi: 10.1017/S0007114508019880.

[5] M. B. Zimmermann *et al.*, “The effects of iron fortification on the gut microbiota in African children: a randomized controlled trial in Côte d’Ivoire,” *The American Journal of Clinical Nutrition*, vol. 92, no. 6, pp. 1406–1415, Dec. 2010, doi: 10.3945/ajcn.110.004564.

[6] X. Guo, X. Xia, R. Tang, J. Zhou, H. Zhao, and K. Wang, “Development of a real-time PCR method for *Firmicutes* and *Bacteroidetes* in faeces and its application to quantify intestinal population of obese and lean pigs,” *Letters in Applied Microbiology*, vol. 47, no. 5, pp. 367–373, Nov. 2008, doi: 10.1111/j.1472-765X.2008.02408.x.

[7] M. C. Collado, M. Derrien, E. Isolauri, W. M. de Vos, and S. Salminen, “Intestinal integrity and Akkermansia muciniphila, a mucin-degrading member of the intestinal microbiota present in infants, adults, and the elderly,” *Appl Environ Microbiol*, vol. 73, no. 23, pp. 7767–7770, Dec. 2007, doi: 10.1128/AEM.01477-07.

[8] U. Ghoshal, R. Shukla, D. Srivastava, and U. C. Ghoshal, “Irritable Bowel Syndrome, Particularly the Constipation-Predominant Form, Involves an Increase in Methanobrevibacter smithii, Which Is Associated with Higher Methane Production,” *Gut Liver*, vol. 10, no. 6, pp. 932–938, Nov. 2016, doi: 10.5009/gnl15588.

[9] A. Fite *et al.*, “Identification and quantitation of mucosal and faecal desulfovibrios using real time polymerase chain reaction,” *Gut*, vol. 53, no. 4, pp. 523–529, Apr. 2004, doi: 10.1136/gut.2003.031245.

[10] H. Fehlner-Peach *et al.*, “Distinct Polysaccharide Utilization Profiles of Human Intestinal Prevotella copri Isolates,” *Cell Host Microbe*, vol. 26, no. 5, pp. 680-690.e5, Nov. 2019, doi: 10.1016/j.chom.2019.10.013.

[11] F. J. Picard *et al.*, “Use of tuf sequences for genus-specific PCR detection and phylogenetic analysis of 28 streptococcal species,” *J Clin Microbiol*, vol. 42, no. 8, pp. 3686–3695, Aug. 2004, doi: 10.1128/JCM.42.8.3686-3695.2004.

**Supplementary Table 2:** Body Composition among study participants

| **Parameter** | **Healthy Controls**  **(n = 18)** | **Endurance athletes**  **(n = 25)** | **Strength athletes**  **(n = 13)** | **ANOVA p-value** | **Cohen’s Effect sizes (η²)** | **Interpretation** |
| --- | --- | --- | --- | --- | --- | --- |
| Trunk fat mass (kg) | 6.91 ± 2.82^†^ | 5.59 ± 2.62 | 7.43 ± 3.64*^‡^* | 0.001 | 0.234 | Large |
| Trunk lean mass (kg) | 21.84 ± 1.88^†^ | 22.68 ± 2.96 | 27.09 ± 6.41*^‡^* | 0.001 | 0.234 | Large |
| Arm fat (Right, kg) | 1.02 ± 0.33^*^ | 0.77 ± 0.32 | 1.07 ± 0.37*^‡^* | 0.009 | 0.141 | Large |
| Arm fat (Left, kg) | 0.73 ± 0.35 | 0.59 ± 0.28 | 0.80 ± 0.32 | 0.147 | 0.035 | Small |
| Leg fat (Right, kg) | 3.26 ± 1.03^*^ | 2.48 ± 1.06 | 3.28 ± 1.25 | 0.010 | 0.137 | Moderate |
| Leg fat (Left, kg) | 2.88 ± 0.95^*^ | 2.25 ± 1.01 | 2.92 ± 1.23 | 0.023 | 0.105 | Moderate |
| Arm lean mass (Right, kg) | 2.90 ± 0.39^†^ | 2.90 ± 0.41 | 3.73 ± .84*^‡^* | 0.004 | 0.176 | Large |
| Arm lean mass (Left, kg) | 2.79 ± 0.40^†^ | 2.76 ± 0.33 | 3.58 ± 0.74*^‡^* | 0.002 | 0.202 | Large |
| Leg lean mass (Right, kg) | 7.96 ± 0.99^†^ | 8.45 ± 0.99 | 9.58 ± 2.09*^‡^* | 0.005 | 0.182 | Large |
| Left leg lean mass (kg) | 7.76 ± .92^†^ | 8.34 ± 0.99 | 9.55 ± 2.20*^‡^* | 0.003 | 0.203 | Large |
| Arm fat (Right, %) | 24.51 ± 5.96^*^ | 19.66 ± 5.97 | 20.93 ± 4.59 | 0.010 | 0.134 | Moderate |
| Arm fat (Left, %) | 19.62 ± 6.73 | 16.31 ± 5.57 | 17.05 ± 4.46 | 0.162 | 0.031 | Small |
| Leg fat (Right, %) | 27.68 ± 6.98^*^ | 21.20 ± 6.72 | 24.08 ± 6.32 | 0.002 | 0.193 | Large |
| Leg fat (Left, %) | 25.71 ± 16.93^*^ | 19.50 ± 6.39 | 22.05 ±6.94 | 0.009 | 0.142 | Large |
| Arm lean mass (Right, %) | 71.19 ± 5.63^*^ | 75.50 ± 5.72 | 74.72 ± 4.52 | 0.021 | 0.109 | Moderate |
| Arm lean mass (Left, %) | 75.93 ± 6.33 | 78.63 ± 5.28 | 78.43 ± 4.33 | 0.232 | 0.017 | Small |
| Leg lean mass (Right, %) | 68.61 ± 6.65^*^ | 74.22 ± 6.33 | 71.96 ± 6.07 | 0.004 | 0.175 | Large |
| Leg lean mass (Left, %) | 70.33 ± 6.58^*^ | 75.42 ± 6.62 | 73.80 ± 6.69 | 0.025 | 0.102 | Moderate |
| ASM/weight (%) | 33.23 ± 3.42^*^ | 35.54 ± 3.01 | 34.92 ± 2.82 | 0.043 | 0.081 | Moderate |
| ASM/BMI ratio | 0.99 ± 0.15 | 1.04 ± 0.09 | 1.05 ± 0.11 | 0.220 | 0.056 | Small |

Values are presented as mean ± standard deviation (SD). Group differences were assessed using one-way analysis of variance (ANOVA). Post-hoc pairwise comparisons were performed using Bonferroni correction. Effect size is reported as eta-squared (η²) and interpreted as small (0.01–0.05), moderate (0.06–0.13), or large (≥0.14). ASM: appendicular skeletal muscle mass; BMI: body mass index.*Significant difference between Control and Endurance groups (p < 0.05), †Significant difference between Control and Strength groups (p < 0.05), ‡ Significant difference between Endurance and Strength groups (p < 0.05)

**Supplementary Table 3:** Bone Mineral Content (BMC) and Bone Mineral Density (BMD) among participants

| **Parameter** | **Sedentary Controls (n=18)** | **Endurance Athletes (n=25)** | **Strength Athletes**  **(n=13)** | ***p-value*** | **Effect size** | **Magnitude** |
| --- | --- | --- | --- | --- | --- | --- |
| Left arm BMC (g) | 162.94 ± 22.84^†^ | 178.24 ± 29.72 | 203.92 ± 40.07 | 0.01 | 0.135 | Moderate |
| Right arm BMC (g) | 174.87 ± 22.95^†^ | 186.43 ± 35.94 | 218.66 ± 48.05 | 0.029 | 0.097 | Moderate |
| Left ribs BMC (g) | 86.50 ± 13.56^†^ | 88.38 ± 16.39 | 108.95 ± 18.19^‡^ | 0.002 | 0.197 | Large |
| Right ribs BMC (g) | 81.93 ± 11.59^†^ | 89.95 ± 14.71 | 102.57 ± 21.61 | 0.003 | 0.198 | Large |
| Thoracic spine BMC (g) | 89.83 ± 16.13^†^ | 104.81 ± 19.56 | 113.85 ± 27.83 | 0.007 | 0.17 | Large |
| Lumbar spine BMC(g) | 54.77 ± 11.24^†^ | 62.54 ± 11.86 | 68.84 ± 15.00 | 0.011 | 0.158 | Large |
| Pelvis BMC(g) | 250.99 ± 58.37^†^ | 292.38 ± 58.22 | 363.71 ± 98.22^‡^ | 0.001 | 0.274 | Large |
| Left leg BMC(g) | 436.99 ± 52.45^*†^ | 526.46 ± 102.79 | 531.69 ± 99.32 | 0.001 | 0.242 | Large |
| Right leg BMC (g) | 431.56 ± 63.23^*†^ | 524.33 ± 98.55 | 522.35 ± 95.70 | 0.001 | 0.221 | Large |
| Subtotal BMC (g) | 1770.32 ± 244.08^*†^ | 2053.49 ± 344.23 | 2234.54 ± 435.13 | 0.003 | 0.187 | Large |
| Head BMC (g) | 553.61 ± 106.81 | 537.22 ± 113.21 | 535.77 ± 118.26 | 0.871 | 0.005 | Trivial |
| Total BMC (g) | 2323.48 ± 308.83^†^ | 2582.70 ± 450.81 | 2793.42 ± 504.99 | 0.013 | 0.153 | Large |
| Left arm BMD (g/cm^2^) | 0.76 ± 0.06^*†^ | 0.82 ± 0.07 | 0.86 ± 0.09 | 0.001 | 0.056 | Small |
| Right arm BMD (g/cm^2^) | 0.80 ±0.06^†^ | 0.86 ± 0.08 | 0.93 ± 0.11^‡^ | 0.001 | 0.061 | Moderate |
| Left ribs BMD (g/cm^2^) | 0.69 ± 0.08^†^ | 0.72 ± 0.06 | 0.81 ± 0.11^‡^ | 0.005 | 0.159 | Large |
| Right ribs BMD (g/cm^2^) | 0.69 ±0.08^†^ | 0.74 ± 0.06 | 0.81 ± 0.12^‡^ | 0.001 | 0.096 | Moderate |
| Thoracic spine BMD (g/cm^2^) | 0.76 ±0.12^*†^ | 0.85 ± 0.12 | 0.92 ± 0.13 | 0.002 | 0.207 | Large |
| Lumbar spine BMD (g/cm^2^) | 0.94 ±0.13^†^ | 1.06 ± 0.15 | 1.12 ± 0.14 | 0.005 | 0.159 | Large |
| Pelvis BMD (g/cm^2^) | 1.13 ± 0.13^†‡^ | 1.20 ± 0.13 | 1.37 ± 0.22 | 0.001 | 0.115 | Moderate |
| Left leg BMD (g/cm^2^) | 1.16 ±0.09^*†^ | 1.33 ± 0.14 | 1.31 ± 0.16 | 0.001 | 0.078 | Moderate |
| Right leg BMD (g/cm^2^) | 1.15 ± 0.09^*†^ | 1.33 ± 0.12 | 1.30 ± 0.17 | 0.001 | 0.07 | Moderate |
| Subtotal BMD (g/cm^2^) | 0.97 ± 0.08^*†^ | 1.08 ± 0.10 | 1.12 ± 0.13 | <0.0001 | 0.079 | Moderate |
| Head BMD (g/cm^2^) | 2.18 ± 0.30 | 2.19 ± 0.38 | 2.13 ± 0.48 | 0.871 | 0.005 | Trivial |
| Total BMD (g/cm^2^) | 1.12 ± 0.09^*†^ | 1.21 ± 0.12 | 1.25 ± 0.13 | 0.005 | 0.033 | Small |

Values are presented as mean ± standard deviation (SD). Group differences were assessed using one-way analysis of variance (ANOVA). Post-hoc pairwise comparisons were performed using Bonferroni correction. Effect size is reported as eta-squared (η²) and interpreted as small (0.01–0.05), moderate (0.06–0.13), or large (≥0.14). ASM: appendicular skeletal muscle mass; BMI: body mass index.

*Significant difference between Control and Endurance groups (p < 0.05)

†Significant difference between Control and Strength groups (p < 0.05)

‡ Significant difference between Endurance and Strength groups (p < 0.05)

**Supplementary Table 4:** Nutrient intake in study participants

|  | **Healthy Controls (n=24)** | | | **Endurance Athletes (n=27)** | | | **Strength Athletes (n=29)** | | | **p-value** | **η²** |
| --- | --- | --- | --- | --- | --- | --- | --- | --- | --- | --- | --- |
|  | **Mean ± SD** | **Median** | **IQR** | **Mean ± SD** | **Median** | **IQR** | **Mean ± SD** | **Median** | **IQR** |  |  |
| **Fatty acids & Lipid Compounds** | | | | | | | | | | | |
| Linoleic acid (C18:2n6) (mg) | 13228.2 ± 3873.6^*†^ | 12199.5 | 3197.5 | 23879.1 ± 7756.8 | 23690.8 | 6297.8 | 20084.8 ± 5987.3 | 20325.7 | 7466.6 | <0.0001 | 0.34 |
| Eicosapentaenoic acid (C20:5n3) (mg) | 555.3 ± 1129.3 | 114.1 | 215.4 | 177.7 ± 283.0 | 56.2 | 165.6 | 419.4 ± 681.5 | 112.1 | 513.3 | 0.555 | 0.012 |
| Docosahexaenoic acid (C22:6n3) (mg) | 66.9 ± 133.6^†^ | 0 | 33.6 | 115.9 ± 120.4 | 94 | 139.1 | 173.6 ± 215.9 | 88.6 | 230.5 | 0.008 | 0.107 |
| α-Linolenic acid (C18:3n3) (mg) | 315.3 ± 218.7^*†^ | 275.9 | 235.6 | 1414.0 ± 1041.8 | 1395.6 | 1468.2 | 1298.4 ± 1087.4 | 1069.2 | 1188.9 | <0.0001 | 0.259 |
| Cholesterol (mg) | 510.8 ± 1138.6^*†^ | 65.2 | 174.9 | 392.1 ± 257.9 | 316.1 | 424.1 | 536.2 ± 361.5 | 484.7 | 476.8 | 0.001 | 0.171 |
| **Fat-Soluble Vitamins** | | | | | | | | | | | |
| Vitamin A, RAE (µg) | 398.3 ± 186.4^*†^ | 419 | 307.4 | 2461.2 ± 2078.4 | 1663.9 | 1303.4 | 2180.0 ± 2107.7 | 1799.1 | 1130 | <0.0001 | 0.467 |
| Vitamin D (D2+D3) (IU) | 75.4 ± 137.4^*†^ | 32.3 | 24.1 | 3005.9 ± 2926.0 | 3245.5 | 4304.5 | 2050.3 ± 2540.6 | 391.3 | 3372.5 | <0.0001 | 0.276 |
| α-Tocopherol Equivalent [VITE] (mg) | 3.2 ± 1.7^*†^ | 3 | 1 | 11.7 ± 5.7 | 10.1 | 7.7 | 9.0 ± 5.0 | 8.3 | 5.9 | <0.0001 | 0.459 |
| Phylloquinones [VITK1] (µg) | 85.7 ± 58.1^*†^ | 66.9 | 44.7 | 378.2 ± 299.1 | 298.7 | 385.6 | 277.7 ± 261.9 | 167.5 | 351.9 | <0.0001 | 0.294 |
| Menaquinones [VITK2] (µg) | 4.3 ± 5.8^*†^ | 2 | 5.3 | 24.7 ± 15.6 | 24.5 | 21.2 | 36.6 ± 26.6 | 31.8 | 46.2 | <0.0001 | 0.399 |
| β-Carotene (µg) | 1511.5 ± 805.0^*†^ | 1344.6 | 1470.2 | 9024.0 ± 7061.2 | 7714.7 | 6653.1 | 7373.4 ± 5870.7 | 5525 | 6311.4 | <0.0001 | 0.362 |
| **Water-Soluble Vitamins** | | | | | | | | | | | |
| Thiamine (B1) (mg) | 1.2 ± 0.3^*†^ | 1.1 | 0.2 | 2.6 ± 0.7 | 2.4 | 0.9 | 2.2 ± 0.8 | 2.2 | 1 | <0.0001 | 0.48 |
| Riboflavin (B2) (mg) | 0.8 ± 0.2^*†^ | 0.7 | 0.2 | 2.0 ± 0.6 | 2 | 0.5 | 1.9 ± 0.6 | 2 | 1 | <0.0001 | 0.503 |
| Niacin (B3) (mg) | 12.6 ± 2.5^*†^ | 12.8 | 2 | 26.7 ± 7.1 | 24.2 | 8.8 | 25.6 ± 8.7 | 23.7 | 11.2 | <0.0001 | 0.531 |
| Pantothenic Acid (B5) (mg) | 5.5 ± 0.9^*†^ | 5.3 | 1.3 | 13.5 ± 3.4 | 13.2 | 5.7 | 13.2 ± 4.3 | 13.2 | 6.4 | <0.0001 | 0.254 |
| Total B6 (mg) | 7.4 ± 25.5^*†^ | 1.3 | 0.6 | 21.9 ± 30.5 | 4.7 | 34.8 | 38.2 ± 57.5 | 5.6 | 52.6 | <0.0001 | 0.409 |
| Biotin (B7) (µg) | 15.8 ± 6.1^*†^ | 13.8 | 7 | 59.5 ± 26.0 | 53.8 | 29.4 | 59.9 ± 32.5 | 50.6 | 33.2 | <0.0001 | 0.505 |
| Total Folates (B9)(µg) | 357.1 ± 169.6^*†^ | 315.3 | 84.3 | 972.8 ± 359.4 | 977.1 | 607.3 | 1029.8 ± 626.6 | 996.4 | 861.9 | <0.0001 | 0.415 |
| Total Ascorbic Acid (mg) | 60.9 ± 29.2^*†^ | 52.1 | 41.1 | 259.0 ± 150.3 | 223.4 | 246.5 | 195.3 ± 133.4 | 170.2 | 158.7 | <0.0001 | 0.392 |
| **Minerals** | | | | | | | | | | | |
| Iron [FE] (mg) | 13.9 ± 4.8^*†^ | 12.7 | 4.4 | 33.5 ± 11.8 | 32.4 | 16.2 | 27.7 ± 11.4 | 28.3 | 15.4 | <0.0001 | 0.382 |
| Magnesium [MG] (mg) | 414.9 ± 99.6^*†^ | 391 | 131.4 | 1113.3 ± 331.9 | 1111 | 474.6 | 907.5 ± 372.4^‡^ | 927 | 509.7 | <0.0001 | 0.219 |
| Selenium [SE] (µg) | 78.4 ± 52.8^*†^ | 66.6 | 54.6 | 198.8 ± 62.0 | 184 | 68.1 | 214.8 ± 76.0 | 202.4 | 117 | 0.003 | 0.508 |
| Zinc (mg) | 14.3 ± 11.8^*†^ | 10.3 | 2.9 | 21.7 ± 4.6 | 21.5 | 8 | 19.9 ± 5.3 | 20.1 | 7.3 | <0.0001 | 0.265 |

Data are presented as mean ± standard deviation (SD), median, and interquartile range (IQR). Group differences were assessed using one-way analysis of variance (ANOVA) for normally distributed variables. Post-hoc pairwise comparisons were performed using Bonferroni correction. Effect size is reported as eta-squared (η²). Nutrient intake was analyzed using NSR–Nutrical software, based on the Indian Food Composition Tables (ICMR–NIN, 2017) and the USDA food composition database.

*Significant difference between Control and Endurance groups (p < 0.05)

†Significant difference between Control and Strength groups (p < 0.05)

‡ Significant difference between Endurance and Strength groups (p < 0.05)

**Supplementary Table 5:** Spearman's correlation coefficients between dietary fiber intake and log-transformed relative abundance of selected gut microbial taxa among study participants.

| Dietary Fiber Variable | Gut Microbial Taxon | Spearman's rho (ρ) | *p*-value |
| --- | --- | --- | --- |
| Total Dietary Fiber | *Enterobacteriaceae* | -0.387*** | <.001 |
| Total Dietary Fiber | *Lactobacillus* | -0.432*** | <.001 |
| Total Dietary Fiber | *Prevotella Copri* | 0.290* | 0.012 |
| Total Dietary Fiber | *Methanobrevibacter smithii* | 0.257* | 0.027 |
| Insoluble Dietary Fiber | *Enterobacteriaceae* | -0.371** | 0.001 |
| Insoluble Dietary Fiber | *Lactobacillus* | -0.419*** | <.001 |
| Insoluble Dietary Fiber | *Prevotella Copri* | 0.263* | 0.024 |
| Insoluble Dietary Fiber | *Methanobrevibacter smithii* | 0.233* | 0.045 |
| Soluble Dietary Fiber | *Enterobacteriaceae* | -0.408*** | <.001 |
| Soluble Dietary Fiber | *Lactobacillus* | -0.428*** | <.001 |
| Soluble Dietary Fiber | *Prevotella Copri* | 0.319** | 0.006 |
| Soluble Dietary Fiber | *Methanobrevibacter smithii* | 0.247* | 0.034 |

Relative abundances were log-transformed prior to analysis. Spearman's rank correlation was used to assess associations between dietary fiber intake and gut microbial taxa. Positive and negative correlation coefficients indicate direct and inverse associations, respectively**.** Statistical significance levels: *p* < 0.05 (*), *p* < 0.01 (**), and *p* < 0.001 (***).
